# Bioprosthetic Heart Valves: Upgrading a 50-Year Old Technology

**DOI:** 10.3389/fcvm.2019.00047

**Published:** 2019-04-11

**Authors:** Kan Yan Chloe Li

**Affiliations:** Institute of Cardiovascular Science, University College London, London, United Kingdom

**Keywords:** antibody-mediated rejection, bioprosthetic heart valves, heart valve tissue engineering, immune injury, structural valve degeneration, transcatheter aortic valve replacement implantation

## Abstract

Prosthetic heart valves have been commonly used to address the increasing prevalence of valvular heart disease. The ideal prosthetic heart valve substitute should closely mimic the characteristics of a normal native heart valve. Despite the development of various interventions, an exemplary valve replacement does not exist. This review provides an overview of the novel engineering valve designs and explores emergent immunologic insights into age-dependent structural valve degeneration (SVD).

## Introduction

The increasing prevalence of valvular heart disease worldwide is a global clinical dilemma, where the demand for interventions is expected to hit 850,000 by 2050 (1). Prosthetic heart valves have been used to address this problem and the two commonly used basic types are: surgically implanted mechanical heart valves (MHVs) and biological heart valves (BHVs, which can be implanted either surgically or by a micro-invasive transcatheter approach) (2). The ideal prosthetic heart valve substitute should closely mimic the characteristics of a normal native heart valve and be durable, implantable with impeccable hemodynamics, and thromboresistant. Alas, this exemplary valve replacement is non-existent as currently available prosthetic valves are flawed (3).

## Engineered Designs

MHVs are composed from manufactured substance with mechanically moving parts and can be grouped into three basic design categories: bileaflet, monoleaflet, and caged ball valves. The long-term durability is the main advantage of MHVs, but their use is limited by substantial risks of thrombogenicity and the requirement of lifelong anticoagulation therapies to prevent formation of blood clots. MHVs may be obstructed by blood clots in an open or closed position causing stenosis and/or insufficiency. The complications associated with MHVs render them less desirable for some patients, especially in: (i) young, injury-prone individuals, (ii) pregnant or menstruating females, and (iii) patients in the developing world, where it can be challenging to carefully monitor anti-coagulation (4, 5).

Over 300,000 surgical heart valve replacement operations are performed worldwide annually, where 40–60% of these operations employ BHVs produced using glutaraldehyde-fixed animal (bovine or porcine) tissue (6). The inauguration of BHVs in 1968 involved using an effective valve replacement therapy as a treatment for heart valve disease. Since then, there has been a substantial shift toward the use of BHVs compared to MHVs because BHVs are: (i) effective and durable, particularly in older patients (>60 years); (ii) do not have thrombogenicity complications and circumvent the problem of anti-coagulation medications (7). Currently, the most frequently used BHVs are those derived from porcine aortic valves and calf-pericardium, which are preserved by glutaraldehyde fixation (3, 5). Glutaraldehyde crosslinking prevents immunogenicity, and other methods such as anti-mineralization can diminish cusp calcification (8). The key advantages of BHVs over MHVs are that they have significantly improved hemodynamic results, lower gradients and larger aortic valve/orifice areas (9). Furthermore, bioprostheses can be delivered using minimally-invasive technique transcatheter aortic valve replacement implantation/replacement (TAVI/TAVR).

Despite the numerous advantages of BHVs, they are not as mechanically robust compared to MHVs and exhibit limited durability in younger patients (particularly those younger than 60 years), which is a current obstacle hindering the progress of bioprostheses. The findings from recent research conducted by Goldstone et al. (10) suggest that MHVs may be a safer option than BHVs for some patients, particularly in younger patients, and should be used more. Their results suggest that BHVs have a significantly greater probability of death in patients between age 40–49, compared to the older age groups (50–69 and 70–79). Although this begins to even out in the early phase for the 50–59 age group, BHVs still have a higher probability of death, whereas in the older population (70–79 years), they are identical. Thus, they concluded that the mortality benefit for patients who received an MHV versus those who received a BHV continued up to the age of 55 and 70 for aortic valve replacement and mitral valve replacement, respectively. Careful consideration of the patient's age is required. Older patients are less likely to outlive the BHV durability, whereas younger patients may prefer to opt for BHVs to avoid committing to lifestyle changes (for example, relying on blood thinners, lifetime commitment to a restricted diet, and routine blood testing). BHVs would be a preferred option for many women who desire to have children.

Modern advancements have resulted in a cutting-edge resilient heart valve—Edwards Lifesciences® Inspiris Resilia^TM^ aortic valve, which incorporates state-of-the-art Resilia^TM^ bovine pericardial bioprosthetic tissue. This valve has been reported to exhibit anti-calcification properties, improved sustained hemodynamic performance and durability, thus reducing the likelihood of reoperation (11, 12). Furthermore, it has a unique expandable frame and can be potentially used for future valve-in-valve (ViV) procedures. However, ViV procedures can increase the occurrence of prosthesis-patient mismatch (13–15). In the case of early degeneration, the Inspiris Resilia^TM^ valve can be replaced with a TAVI valve, thus avoiding open heart surgery (16). The 2-year outcomes of the ongoing COMMENCE trial (which aims to evaluate the safety and effectiveness of the Inspiris Resilia^TM^ valve) revealed no SVD observations in any of the 689 patients, and there was ameliorated breathlessness, augmented effective orifice area, diminished mean gradient, and lower paravalvular leak rate (12). The results of the COMMENCE trial will be monitored carefully over the next 6 years, which will provide better insight on the long-term durability of the Inspiris Resilia^TM^ valve.

Since the first successful in-patient TAVI in 2002 performed by Cribier, TAVI has become the preferred procedure (as an alternative to open heart surgery) worldwide for high-surgical-risk patients with severe symptomatic aortic stenosis (AS) (17–19). The main advantages of TAVI is that the micro-invasive TAVI procedures have diminished invasiveness compared to minimally-invasive procedures, and can be performed on the live, beating heart without the need for extracorporeal circulation or cardiopulmonary bypass nor aortic cross clamp. This renders TAVI favorable than MHV and BHV prostheses as it overcomes the requirement for temporary cardiac arrest, and improves patient recovery time (20). Another advantage of TAVI is that the micro-invasive procedures (commonly the transfemoral approach) can be conducted in a completely percutaneous fashion and with local anesthesia only, whereas minimally invasive approaches (such as mini-sternotomy or mini-thoracotomy) for conventional heart valve prostheses necessitate skin incision and general anesthesia (17). Successful TAVI may reduce the requirement for future hospitalization, but the expensive cost of the procedure (circa £18,000) and the requirement of multidisciplinary team and substantial equipment are important considerations (21). Although TAVI has several advantages, the technology can result in the following complications (which can ultimately lead to mortality): (i) conduction defects, for example, atrioventricular block and left bundle branch block (22, 23); (ii) permanent pacemaker implantation—reported to occur in 8.5–25.9% of patients undergoing TAVI within a month after the procedure (24–28); (iii) paravalvular regurgitation (reported to affect circa 10% of patients) due to incomplete apposition of the prosthesis with the aortic annulus, or incorrect implantation depth (29–31) (iv) bleeding and vascular complications; (v) stroke, and (vi) myocardial infarction (22).

Initially, TAVI was performed in high-surgical-risk AS patients, but has gradually expanded to intermediate-surgical-risk patients in both European and American guidelines based on three major trials: SURTAVI (28), NOTION (32), and PARTNER 2 (27). The additional challenge of long-term valve durability arises when attempting to expand TAVI to younger, low-risk patients, and is crucial to tackle due to the longer life expectancy (33). A recent meta-analysis involving 3,484 patients conducted by Witberg et al. (34) found that TAVR appears to be associated with elevated mortality risk. Despite this study's limitations [(i) small patient population; (ii) studied a mixture of low-intermediate-risk patients instead of entirely low-risk; and (iii) predominant use of first generation TAVI devices], SAVR should continue being the choice of treatment for low-surgical-risk patients until more data is acquired. There are ongoing trials [PARTNER 3 (NCT02675114), Evolut R low risk (NCT02701283), and NOTION 2 (NCT02825134)] aiming to extend the use of TAVI to low-risk patients (34, 35) by evaluating the risk/benefit profile of TAVR. The outcome of these trials will provide further insight to whether TAVR should be expanded to low-risk patients.

## Structural Valve Degeneration

With time, the function of many glutaraldehyde-fixed BHVs (GBHVs) begin to deteriorate. This is known as structural valve degeneration (SVD), which occurs over 1–2 decades for elderly patients, attributed to inflammatory/immune response and calcification (36, 37). Calcification can cause valve obstruction, resulting in stenosis and leaflet tearing due to the weakening of tissues, and ultimately detachment of the leaflets, rendering the valve dysfunctional. Thus, younger patients are generally treated with MHVs despite their risks because of the age-dependent BHV degeneration. The development of an efficacious prosthetic heart valve that can be used in younger patients that does not require anticoagulants will be a major advancement in this field.

Our knowledge of the mechanism(s) of age-dependent SVD is incomplete, but it is known to be a multifaceted process comprising an induced immunologic response to bioprostheses (38). Firstly, it can be related to stress and mechanical injury as calcification occurs in areas where stress or strain on the tissue is greatest. The most common mechanism of SVD of mitral bioprostheses is regurgitation as an effect of leaflet prolapse or perforation. Degeneration of aortic bioprostheses usually causes AS (39). Tissue calcification (passive or phospholipid-directed) of fixed biological tissue is believed to occur passively in the bloodstream by recruitment processes taking advantage of calcium binding to tissue-fixed negatively-charged phospholipids. This led to the development of anti-calcification treatment over the past two decades, designed to remove phospholipids. Lipid insudation and inflammation is also a mechanism of age-dependent SVD. Finally, antibody-dependent tissue calcification is a critical mechanism, which will be discussed later.

Anti-calcification processing is a technology that has been employed in the clinic and previous studies have reported their preferred anti-calcification methods. Carpentier et al. (40) reduced the phosphate content in the tissue by blocking calcification binding sites with magnesium ions and/or with a surfactant (Edwards XenoLogic Tween 80, Ethanol) to reduce the rate of calcification. Vyavahere and colleagues studied glutaraldehyde-crosslinked porcine aortic valve cusps and found that ethanol (St. Jude α-Oleic acid) pretreatment of BHV cusps significantly altered the material, rendering it unfavorable for lipid adsorption (41). Jones et al. (42) assessed the effects of several surfactants, where they found that sodium dodecyl sulfate (Medtronic T6) significantly diminished calcification and only in porcine aortic valvular bioprostheses. Post-fixation processing to remove phospholipids has been widely adopted in current commercial BHVs. Importantly, there is no flawless animal model and these previous studies have been performed in juvenile sheep (43, 44), where they fixated on the valve calcification and neglected valve inflammation. Although anti-calcification processing significantly reduces calcification in animal models (such as sheep in these studies), it does not diminish the incidence of SVD in younger patients. Thus, it has not been successfully translated or made the same beneficial impact into the clinic to resolve early SVD (10, 45).

The success of clinical cardiac xenotransplantation has been hindered by the immunogenicity predicament. Although glutaraldehyde fixation is established to reduce immunogenicity and degeneration of heart valve prostheses, immunogenicity (which may trigger calcification) is not abolished (6). This hurdle exists because fixation eliminates the immunogenicity of protein antigens, but, immunogenic xenogeneic carbohydrate antigens persist (6, 7). It is well-established that the paramount antigen is galactose-α1,3-galactose (Gal), which stimulates xenograft rejection of tissue organs from pigs or cows by non-human primates/humans (46). Humans and Old World non-human primates (NHP) do not synthesize α-Gal. Instead, they synthesize large quantities of anti-Gal antibodies, which recognize the Gal antigens on the surface of pig cells (47–49). This immune response results in hyperacute immune xenograft rejection (50).

Lila et al. (7) compared the calcium content of α-Gal-positive and GTKO pig pericardium after subcutaneous implantation during 1 month and identified that α-Gal antigens play a role in BHV calcification. Pericardium that was fixed only with glutaraldehyde exhibited significantly lower calcification levels in GTKO pigs compared with that of α-Gal-positive pigs. Furthermore, glutaraldehyde-fixed pig pericardium decreased calcification levels after formaldehyde, ethanol and Tween 80 (FET) treatment (7). Lila et al. demonstrated that glutaraldehyde-fixed pig pericardium followed by FET treatment and pre-incubation with human anti-Gal Abs exhibited remarkably higher α-Gal-positive pig pericardium. Thus, anti-gal antibody (present in all humans) promotes calcification of wild-type tissue, but not GTKO tissue, even after treatment with anti-calcification processing (7). Another finding was that glutaraldehyde-fixed pig pericardium followed by FET treatment and pre-incubated with human anti-Gal antibodies resulted in significantly increased calcification levels in α-Gal positive pig pericardium compared with GTKO pig pericardium. Thus, there is ample evidence that Gal antigens are a main immune barrier to xenotransplantation (51) and are present on commercially-available GBHVs causing accelerated calcification and degeneration of GBHVs (38, 52–54), and it is likely that the gal antigen is present on TAVIs. Therefore, TAVIs are not tackling the age-dependent SVD problem, nevertheless, they have the advantage of micro-invasive percutaneous placement. Gal antigens can be eliminated by genetic engineering and there is growing interest in utilizing Gal-free animal tissues from GTKO pigs to develop BHVs and other biomedical materials as they would be unaffected by anti-Gal antibody-mediated injury (55).

## Immune Injury

A study by Manji et al. demonstrated that steroid treatment can substantially reduce BHVs calcification and rejection (56). Zilla et al. identified higher levels of calcification in bioprosthetic tissue exposed to graft-specific antibodies (6). Rabbits were immunized with bioprosthetic aortic tissue homogenates to generate high-titer graft-specific antibody. Using a subcutaneous implant model, Zilla et al. demonstrated that the binding of graft-specific antibody to glutaraldehyde-fixed tissue enhanced the level of calcification by circa three times. These results suggest that antibody-mediated inflammation may contribute to BHV calcification and that the antibody originates from a carbohydrate source as the tissue is heavily fixed with glutaraldehyde to eliminate the antigenicity proteins via cross-linking. Several studies have generated alpha-1,3-galactosyltransferase-1 (GT1) knockout (GT1KO) pigs by mutating pigs' alpha-1,3-galactosyltransferase-1 (GGTA-1) to abolish the enzyme function, where the synthesis of α-Gal moieties on glycoproteins is blocked when homozygous (37–39). The tissue from GTKO pigs do not bind to anti-Gal antibody and no specific anti-Gal antibody response is elicited when GTKO cells, tissue, and organs are transplanted (37–39). Importantly, removal of this antigen did not eliminate GTKO xenograft rejection, therefore suggesting that a predominantly antibody-mediated process directed to non-Gal antigens is present (40–43).

Previous studies involving heart or kidney xenotransplants from GTKO pigs into baboons demonstrated that graft rejections were mediated by antibodies bound to non-Gal antigens and complement activation (57, 58). This suggests that some residual xenogenic (porcine) carbohydrate antigens exist and are reactive in primates, even in the absence of the Gal antigen (59–63). The additional two immunogenic endothelial cell carbohydrate antigens that have been identified are the Hanganutziu-Deicher (H-D) or N-glycolylneuramic acid (Neu5Gc) antigen and the Sid blood group antigen (Sd^a^)/CAD (produced by the porcine β1,4N-acetylgalacto-saminyl transferase 2 (B4GALNT2) gene) (64, 65). This carbohydrate is presented on the human embryonic kidney cells with pig B4GALNT2. However, B4GALNT2 is present in both human and primate. Further study is then required to delineate the structure of these carbohydrates (59, 65). The oligosaccharide Neu5Gc is expressed in all mammals apart from humans and New World monkeys. A deletion in the *CAMH* gene encoding the enzyme cytidine monophosphate-*N-*acetyl-neuramic acid hydroxylase (CMAH), which is involved in sialic acid synthesis, disables humans from producing Neu5Gc (66–69). Thus, humans synthesize natural anti-Neu5Gc antibodies (69–72), whereas Neu5Gc is present on pig valves and pericardium (73, 74). This antibody reactivity is predicted to contribute to clinical xenograft rejection, but it remains challenging to confidently determine its impact due to the lack of anti-Neu5Gc antibody in experimental NHP models (75). Reuven et al. demonstrated the expression of Neu5Gc in native cardiac tissues and in six commercial BHVs (76). It is possible that removal of this polysaccharide may reduce the antibody response and calcification. The most recently identified xenogenic antigen is the Sd^a^ blood group, which is predominantly expressed in human gastrointestinal epithelial cells and at widely variant levels in human red blood cells and other tissues and fluids. Recently, the porcine B4GALNT2 locus has been genetically engineered and it has been found that there are preformed NHP and human antibodies to B4GALNT2-dependent antigens (75).

Collectively, antibody reactivity to the three aforementioned major xenogeneic glycans, GGTA-1 (Gal), CMAH (Neu5Gc), and B4GALNT2 (Sd^a^) account for the majority of preformed human anti-pig antibody reactivity (75, 77). Importantly, it has been found that pigs engineered with mutations that abolish the production of the three known xenogenic glycans (triple knockout), exhibit the lowest level of antibody reactivity in both human and baboon serum (65, 77). However, there may be more immunogenic glycans and proteins which are yet to be discovered. Knowledge of all the residual non-Gal antigens could revolutionize the field of xenotransplantation by circumventing the immunogenicity predicament.

## Conclusion

Transcatheter valves are currently used in the clinic with over 100,000 TAVI procedures performed worldwide in the past decade (33). Although they possess the advantage of percutaneous placement, their efficacy and durability are not well-established as they have only been briefly active. Nevertheless, TAVI should be considered as the biggest breakthrough in cardiac surgery technology since the last five decades and it is shifting the paradigm toward micro-invasive cardiac surgery. However, one may consider transcatheter valves to be basic in terms of their tissue because their leaflets are like currently available surgical bioprostheses by continuing to employ the basic technology of chemical fixation of bovine or porcine tissues meaning they are still subject to SVD. Furthermore, these valves still utilize dead (non-vital) tissue, ergo any mechanical, chemical, or immunologic damage will persist and exacerbate over time as they current cannot repair themselves or regenerate.

Currently available prostheses are flawed and their use is further restricted by lacking the capability of growth, repair, remodeling, and regeneration (78). Non-regenerative mechanical and biological prostheses fail to integrate, remodel, or grow. This renders them unideal, particularly in pediatric patients who will have to undergo multiple surgeries and intervention redoes throughout their life, with an augmenting risk of morbidity and mortality, and exacerbated quality of life. This has been the driving force of a relatively novel field of research known as heart valve tissue engineering in which early approaches have concentrated on developing new viable tissue via scaffold engineering, cells, and growth factors with the capability to grow in the body via use of a matrix that is seeded *in vitro* with harvested cells (13, 79). Tissue engineered heart valves (TEHVs) are flexible (can adapt and grow with age), non-thrombogenic, non-immunogenic, durable in all age groups (not just in older groups unlike BHVs, which will eliminate reoperations), and have ameliorated cellular viability making them advantageous over MHV and BHVs. However, several shortcomings (financial, logistical, and technical) must be overcome to bring safe, feasible TEHVs into clinical practice.

## Author Contributions

The author confirms being the sole contributor of this work and has approved it for publication.

### Conflict of Interest Statement

The author declares that the research was conducted in the absence of any commercial or financial relationships that could be construed as a potential conflict of interest.
